# The Scientific Investigation of Dental Materials

**Published:** 1920-04

**Authors:** 


### THE SCIENTIFIC INVESTIGATION OF DENTAL MATERIALS

The *Dental Cosmos* publishes in its March issue a report from the National Bureau of Standards on certain phases of the amalgam problem. The report is voluminous and technical and, like analogous reports of scientific investigations relative to dental problems, will be of interest to but a limited number of readers. To students of the chemistry and metallurgy of amalgams and amalgam alloys it will contain matter of absorbing interest, dealing as it does with the fundamental principles which characterize the formation of these important alloys, and emanating from such an acknowledged authoritative extra-dental source, unbiased by personal, professional or commercial interests, it is sure to command the thoughtful attention and consideration of specialists in this particular field.

When in 1895 we published the pioneer investigations of Dr. G. V. Black on the same topic we were favored with a volume of criticism, friendly and otherwise, to the effect that studies of that type were "too scientific" for the average reader; it was "over their heads" and much more to the same purpose. But if it was "caviar to the general," it was solid intellectual nourishment to those who realized its significant importance, and when in due course the principles elucidated by Dr. Black came to be interpreted in terms of practice, then the value of his pioneer studies became clearly evident to all who appreciated the general improvement in amalgam alloys thus made available to the whole dental profession.

The same state of affairs characterized the publication of the work of Miller on the origin of dental caries and the human mouth as a focus of infection. As reports of scientific laboratory research Miller's papers were read by the few who were interested in his field of study from the scientific standpoint. But when his generalizations came to be applied in practice as the foundations of oral hygiene, the prophylaxis of tooth decay and the treatment of systemic disorders arising from dental focal infections, the dental profession of the entire world awoke to a realization of the fundamental importance of subjecting these dental problems to the precise methods of investigation of scientific research work.

The failure to comprehend the value of scientific research work by the self-styled ''practically-minded'' type of individual is mainly due to his lack of understanding of the purpose and character of scientific research and of the difference between pure science and applied science.

Pure science seeks by precise methods to determine the truth by elimination of error and to thus discover the relation of cause and effect in certain orders of phenomena. Applied science is the utilization of the underlying principles or natural laws governing phenomena in their application to the needs of man as expressed in the arts and manufactures.

Applying these considerations to the case in point, the work of Black and his contemporaries on the amalgams left much to be determined by subsequent investigation. Moreover, before the work of any investigator can be accepted as a final exposition of the truth it must pass the crucial test of revision by other equally competent investigators. Many questions bearing on the composition, treatment and behavior of amalgam alloys have arisen as the result of practical experience in their manufacture and use since the earlier investigators published their findings. Such questions have been demanding settlement to the end that a higher degree of perfection and greater satisfaction in the practical use of these important alloys may be attained.

It is therefore matter for congratulation that the National Bureau of Standards has entered upon this work and has given earnest of its intention to continue it in directions relating to dental materials other than amalgams.

It is manifest that the interest of our National government in this important field is one of the many outgrowths of the war experience during which the government was a large consumer of dental material to meet the requirements of its army and navy dental corps.

The day of empiricism in connection with these materials and their use is rapidly passing. The practical interest of our government in placing the development and production of all of its material requirements upon a scientific basis is rapidly sounding the death-knell to empiricism and rule of thumb methods in all departments of the arts and manufactures. So far as dentistry is concerned the result is one devoutly to be desired, and the *motif* which is manifest in the amalgam report by the National Bureau of Standards should be accepted as one more of the many impulses, and a most potential one, which is helping to develop dentistry as a scientific profession.

